# Malignant Transformation of Meningiomas

**DOI:** 10.7150/jca.105024

**Published:** 2025-02-10

**Authors:** Jinxiu Yu, Jiaojiao Deng, Leihao Ren, Lingyang Hua, Ye Gong

**Affiliations:** 1 Department of Neurosurgery, Huashan Hospital, Shanghai Medical College, Fudan University, Shanghai, China.; 2 Institute of Neurosurgery, Fudan University, Shanghai, China.; 3 Shanghai Key Laboratory of Brain Function Restoration and Neural Regeneration, Fudan University, Shanghai, China.; 4 Department of Critical Care Medicine, Huashan Hospital, Shanghai Medical College, Fudan University, Shanghai, China.

**Keywords:** Meningioma, Malignant transformation, Secondary meningioma, Atypical meningioma, Malignant meningioma

## Abstract

Meningioma is the most common intracranial tumor. Sometimes, meningiomas can develop malignant transformation (MT). In this review, we review the incidence of MT of meningiomas. The incidence of MT of grade 2 meningiomas is likely to be higher than benign meningiomas. Approximately 1% to 4% of WHO Grade 1 meningiomas may undergo MT, while about 26% to 33% of Grade 2 meningiomas experience MT. Time to MT of grade 2 meningiomas seemed to be shorter than MT of grade 1 meningiomas. The time for Grade I meningiomas to undergo MT is approximately 5 years, while Grade II meningiomas typically experience MT in about 3 years. Several risk factors may be associated with MT, including non-skull base location, high mitotic Index, a larger primary tumor size, shorter recurrence time interval and male. Potential molecular mechanisms of MT include chromosomal abnormalities (Chromosome 22q deletion, NF2 gene mutation, loss of chromosome 1p), genomic alterations (FOXM1, CDKN2A/B and TERTp), and meningioma cancer stem cells. Secondary meningiomas may have poor tumor control rates and overall survival rates than primary meningiomas. Besides, the role of radiotherapy in MT of meningiomas is unclear. Major concerns are whether radiotherapy can induce MT of meningiomas, and whether radiotherapy can prolong time to MT through long term control of meningiomas. This review summarizes the MT of meningiomas, and may provide the direction for further study of meningiomas.

## Introduction

Meningiomas originate from meningothelial cells, are the most common primary intracranial tumors, and account for almost 39.0% and 54.5% of all and non-malignant central nervous system (CNS) tumors respectively 1. According to WHO classification, meningiomas are divided into grade 1 (benign), 2 (atypical), and 3 (anaplastic). From the CBTRUS statistical report in the United States in 2014-2018, of meningioma with documented WHO grade (65.7%), 35.9%, 8.2% and 0.7% were WHO grade 1, 2 and 3 respectively. 1 Grade 1 meningiomas are usually relatively indolent and slowing tumors that display decelerated growth during tumor enlargement 2. High grade meningiomas usually grow faster and are likely to recur.

**Figure 1:**
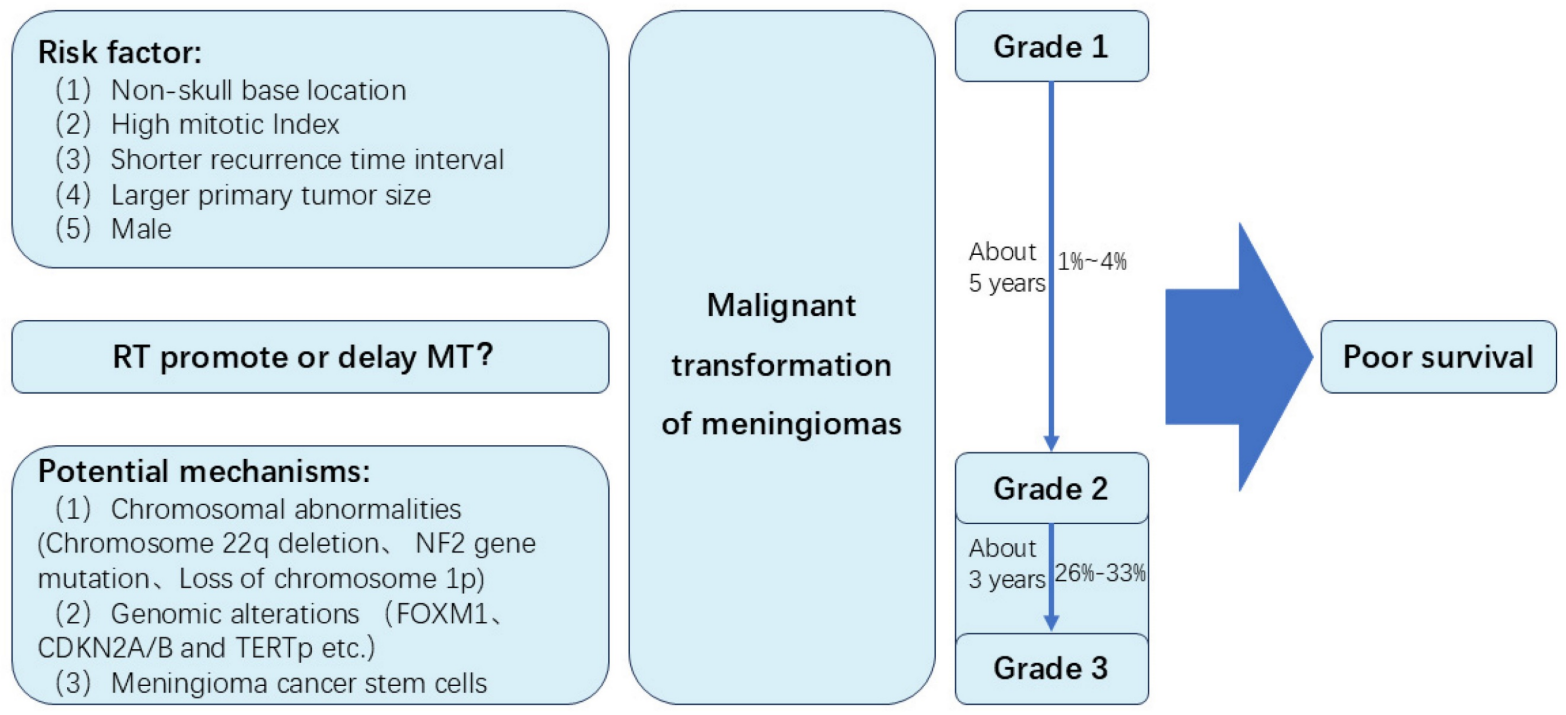
Summary of the malignant transformation of meningiomas in this study

The management of meningiomas includes surgical resection, radiotherapy, systematic therapy and observation. For most of symptomatic or enlarging meningiomas, surgery is the primary treatment 3. Extent of resection (EOR) is a very important prognostic factor. However, event after gross total resection, meningiomas can recur. Approximately 20% of benign meningiomas are likely to be recurrent and invasive in fact 4. Nearly 30%-40% of atypical meningiomas can recur 5. Sometimes these recurrent lesions can transform to higher WHO grade, which is so-called malignant transformation (MT). In 1958, Hoffmann *et al.* 6 reported an autopsied case of a meningioma with MT and implantation in the subarachnoid space after periodic surgical removal over a period of 10 years. Since that, a small number of MT cases have been reported in many retrospective studies. However, many issues remain to be addressed, including the incidence, time to MT, risk factors, potential molecular mechanism and prognosis of MT. In this review, we will summarize some of the most recent advances of MT (Figure 1).

## Evolution of WHO Classification

Meningiomas have been classified according to WHO grading system since 1993 7. The WHO classification has been revised in 2000 [8], 2007 [9], 2016 [10] and 2021 11. In 2000, more specific criteria for grade 2 and 3 had been defined. Either of the following should be diagnosed as grade 2 meningiomas: (1) 4-19 mitoses per 10 HPF; (2) three or more of the following: increased cellularity, small cell change, prominent nucleoli, pattern less or sheet-like growth, foci of spontaneous or geographic necrosis 8. And either of the following should be diagnosed as grade 3: (1) ≥20 mitoses per 10 HPF; (2) anaplastic (malignant) cytology resembling that of carcinoma, melanoma, or high-grade sarcoma 8. In the revision of the 2007 WHO classification, predominant chordoid or clear cell morphology, and predominant papillary or rhabdoid morphology were added into the diagnosis of grade 2 and 3 respectively 9. In 2016, brain invasion was added as a standalone diagnostic criterion for grade 2 meningiomas 10. In 2021, molecular markers had been added as diagnostic criteria for selected subtypes. KLF4/TRAF7 mutations can be diagnosed as secretary meningiomas. SMARCE1 mutation was associated with clear cell meningiomas. Particularly, any meningioma with CDKN2A/B homozygous deletion or TERT promoter mutation should be diagnosed as WHO grade 3, regardless of histological criteria of anaplasia. Furthermore, papillary and rhabdoid subtypes were no longer allotted to grade 3 in absence of other criteria 11.

At present, there were some flaws in the molecular and histological diagnosis of meningiomas, including spatial and longitudinal heterogeneity of mutations, and subjective interpretation of histological criteria. These problems may be overcome by DNA methylation-based subtyping of meningioma, which may be superior to candidate gene panel sequencing and WHO classification for predicting time to tumor recurrence 12-15. With growing knowledge of molecular diagnosis of meningioma, the WHO classification will continue to change. With each revision of WHO classification, the proportion of grade 2 and 3 meningiomas will have a little change.

## Incidence of malignant transformation

Prior to the 2000 WHO classification, it had been reported almost 1-2% of grade 1 meningiomas transformed to high-grade meningiomas 16, 17. Based on the 2000 WHO classification, Schiffer *et al.* 18 and McGovern *et al.* 19 reported nearly 4% of benign meningiomas progressed to grade 2 or 3 meningiomas. In the studies of Champeaux *et al.* 20 and Yeon *et al.* 21, 2.2% and 2.7% of grade 1 meningiomas exhibited MT according to the 2016 WHO classification respectively. It was unclear that whether these different proportion of MT were ascribed to the different version of WHO classification or the increasing use of radiotherapy. Therefore, in a recent systematic review and meta-analysis of MT of WHO grade 1 meningiomas, Nakasu *et al.* 22 reported 56 and 24 cases of MT were found in 2639 patients from surgery group and 5969 patients from radiosurgery group respectively. The incidence of MT was 2.98/1000 and 0.50/1000 patient-years in surgery and radiosurgery group respectively. However, due to a higher proportion of skull-base tumors and a lower proportion of reoperation for recurrent tumors, the incidence of MT was underestimated in radiosurgery group.

It was reported 26%-33% of atypical meningiomas occurred anaplastic transformation based on histological analysis at the time of recurrence 17. Of those recurrent meningiomas, it was reported 6.3%-41.4% 16, 21, 23-25 could transform to higher grade tumors. During the history of malignant meningiomas, 14%-42.9% of cases were initially diagnosed as low-grade tumors 26. As a higher recurrent rate and more aggressive behavior, the incidence of MT of grade 2 meningiomas is likely to be higher than benign meningiomas.

## Time to malignant transformation

MT is a time-dependent event. In the study of Nakasu *et al.*, 22 the median time to MT of WHO grade 1 meningiomas was 5 years (IQR, 2.5-8.2). Younger patients had a longer time to MT. For elderly patients (>50 years), time to MT was limited by life expectancy. The cumulative incidence curve of MT indicated a nearly linear increase in the first 8-9 years and a slower increase thereafter in the younger patients (≤50 years).

The median time of MT of grade 2 was about 3 years in most studies. 25-28 Kwon SM *et al.* 27 reported 9 cases of atypical meningiomas underwent MT to anaplastic meningiomas. The median time to MT was 19 months (range, 7-78). In the studies of Al-Mefty O *et al.* 25 and Yang *et al.* 28, the median time of grade 2 was 37 months (range, 12-82) and 39.8 months (range, 13.5-62.5) respectively. However, in these two studies, only 3 cases of MT patients were reported in each study. A relatively large number of MT cases was reported by Champeaux *et al.* 26, 50 cases of atypical meningioma underwent MT to anaplastic meningiomas with median time of 3.2 years (IQR, 1.2-4.9). The time to MT of grade 2 was approximately 3 years according to previous studies. It seemed to be shorter than MT of grade 1 meningiomas. This may be due to rapid tumor growth speed and short recurrent time in grade 2 meningiomas.

## Risk factors associated with malignant transformation

### Non-skull base location

Non-skull base meningiomas may be more prone to malignant transformation. In the study of Nakasu *et al.* 22, skull-base tumor location was significantly associated with MT of WHO grade 1 meningiomas, the higher proportion ofw skull-base location, the lower incidence of MT. McGovern *et al.* 19 revealed a similar result, patients with non-skull base meningiomas were likely to occur MT (36%) compared with patients with skull base meningiomas (5%, p=0.024). Due to a higher genomic instability and proliferative potential, 29-31 non-skull base meningiomas usually have different regrowth pattern and behavior. Skull base meningiomas often have a lower rate and plateau pattern of regrowth, while non-skull base meningiomas continue to grow 19, 29, 32. Non-skull base meningiomas were significantly related with a higher WHO grade and a higher index of Ki67 33. Therefore, skull base meningiomas often have a relatively indolent nature history and a lower incident rate of MT.

### High mitotic Index

The mitotic index is typically defined as the percentage of cells in mitosis (M-phase) within a specific tissue or cell population. This metric serves as a crucial indicator of aggressiveness and a high potential for proliferation 34-36. Mitotic Index is one of the most important predictors of recurrence in meningiomas 37. Kwon *et al.* 27, 38 conducted research to investigate the clinical factors that could predict the probability of MT of meningiomas. They found an increased mitotic index was the only significant predictor of MT of benign or atypical meningiomas. Nevertheless, variations in quantifying mitotic figures within 10 high-power fields (HPF) small unit areas arise due to subjective factors.

### Shorter recurrence time interval and larger primary tumor size

After analyzing various clinical and radiological factors between the MT group and the non-MT group, researchers found that the recurrence interval for Grade 1 meningiomas that underwent MT was shorter than that of the non-MT group. Furthermore, when comparing the recurrence intervals of Grade 2 meningiomas, researchers discovered that the recurrence interval in the MT group was similar to that of Grade 2 meningiomas. These findings suggest that MT should be considered in patients with rapidly growing tumors following initial treatment 21. Furthermore, a larger primary tumor size was found in high grade transformation group 21. Tumor size had consistently been identified as a significant risk factor for recurrence 39, 40. Larger tumors presented greater challenges for complete resection and may demonstrated a higher proliferative capacity of tumor cells. Given that meningioma progression results from multiple genetic mutations 25, the likelihood of genetic alterations leading to MT increases in highly cellular tumors.

### Male

Meningiomas exhibit a higher prevalence in females compared to males. Prior epidemiological and pathological studies had demonstrated a link between female sex hormones and the propensity for meningioma formation 41-43. Female/male ratio were 2.3:1 in non-malignant meningiomas 1, while male seemed to be a little predominance in grade 2 and 3 meningiomas 23, 44-47. Some studies indicated a male predominance in the secondary meningiomas. Moliterno *et al.* 48 found a male predominance among the progressed group (64% VS 30%, p=0.04). Peyre *et al.* 49 and Krayenbuhl *et al.* 44 reported male patients prevailed in secondary anaplastic meningiomas. In the study of Sahm *et al.* 50 male was predominance in the TERT mutant cases (10/16) and associated with recurrence. However, in the study of Nakasu *et al.* 22 gender had no effect on the incidence rate of MT of grade 1 meningiomas in surgical series by meta-regression (p=0.088). Reports from the Nationwide Brain Tumor Registry of Japan indicated that male sex was associated with early recurrence of WHO grade I meningiomas after surgical resection. The gender-specific disparities in meningioma tumor behavior are presently not well-defined, necessitating additional examination of the underlying mechanisms. Due to the heightened prevalence of high-grade meningiomas in males and their potential connection to malignant transformation, special consideration may be warranted for male patients.

## Potential molecular mechanisms of malignant transformation

### Chromosomal abnormalities

Chromosome abnormality is one of the most important mechanisms of human malignant tumors, and has been an important part in progressive and recurrent meningiomas. Karyotypic abnormalities and copy-number alterations were associated with tumor aggressiveness 51, 52. Chromosome 22q deletion is the most frequent chromosome abnormality in meningiomas. It was reported chromosome 22q occurred in more than half of meningiomas 53. Deletion of chromosome 22q often occurred in the region of neurofibromatosis type 2 gene (NF2), leading to the occurrence of meningiomas 54. Lots of studies revealed loss of gene function of NF2 was linked to the development of ependymomas, schwannoma and malignant mesothelioma 55-57. Basic research suggested NF2 promoted contact inhibition and tumor suppression by inhibiting mitotic signaling in the cell cortex 58. As a consequence, the inactivation of NF2 takes part in early oncogenic events. Genomic profiling revealed NF2 gene mutation was associated with chromosome instability and was an early and frequent event in MT meningioma samples 59. However, the question was whether NF2 contributed to chromosome instability in meningiomas or whether NF2 loss was the consequence of an earlier event responsible for chromosome instability 59. Some studies reported that the frequency of NF2 gene mutation was similar between benign and high grade meningiomas, indicating NF2 might not associated with progression of meningiomas 60.

Loss of chromosome 1p was associated with MT of meningiomas. Chromosome 1p harbored tumor suppressor genes associated with the malignant progression of meningiomas 61. In malignant transformed meningiomas, 1p loss of heterozygosity and high methylation of the p73 promoter were detected, but were not detected in lower grade primary tumors 62. The rate of deletion of chromosome 1p was increasing with WHO grade: 13%-26% in grade 1 meningiomas, 40%-76% in grade 2, 70%-100% in grade 3 63. Maas SLN *et al.* revealed the progression risk of WHO grade 1 meningiomas was significantly higher in cases exhibiting concurrent 1p/22q deletions (involving 6% or more of the chromosomal arms) than in cases without deletions or with only single 1p/22q deletions 64. Analysis of chromosome 1p can provide an independent and cost-effective biomarker for identifying cases with a higher risk of recurrence 65.

### Genomic alterations

Cell cycle dysregulation is associated with excessive tumor cell growth and proliferation, which could contribute to tumor recurrence and progression. Forkhead box protein M1 (FOXM1) is a master transcription factor for tumor cell growth and proliferation, which is related with several malignant tumors, including glioma, prostate cancer and hepatocellular carcinoma 66-68. FOXM1 could promote mitotic progression by accelerating G1/S and G2/M transition and involve in meningioma progression 69, 70. FOXM1 was associated with higher grade and recurrent meningiomas, and had shorter PFS 70. FOXM1 was upregulated in premalignant grade 1 meningioma years before the grade 3 transformation 71. Cyclin-dependent kinase inhibitor 2A/B (CDKN2A/B) encodes p16INK4A, p14ARF and p15INK4B. p16INK4A and p15INK4B could prevent S-phase entry by inhibiting the CDK4/cyclin D complex. p14ARF could prevent cell proliferation in G1 phase 72. CDKN 2A/B locus loss on 9p was the most frequent recurrent genomic alterations progressing to grade 3 59, and associated with poor prognosis in meningiomas 73. Telomerase reverse transcriptase promoter (TERTp) mutations could promote cell immortalization and proliferation by preventing telomere shortening, and could enhance malignant behavior leading to poor prognosis 74, 75. Researchers found acquisition of an activating TERTp mutation could lead to MT of meningiomas 76-78. According to the literature, the incidence of TERT promoter mutations in meningioma patients with malignant histological progression was as high as 28% 79. In addition, some patients exhibited histological progression, but TERT expression did not increase. This observation suggests that alternative mechanisms, such as alternative lengthening of telomeres, may be associated with telomere maintenance in meningiomas, similar to the situation observed in gliomas 80. Some other gene mutations that are also associated with the MT of meningiomas, including TOP2A、BIRC5 and MYBL2 71.

## Meningioma cancer stem cells

Cancer stem cells (CSCs) represent a subpopulation within tumors that possess the ability for self-renewal and differentiation, functioning as crucial markers of tumor growth, metastasis, and treatment resistance 81. Meningiomas are known to harbor CSCs, which are highly resilient and utilize deregulated stem cell expression profiles, thereby contributing to tumor recurrence, treatment resistance and MT 82-84. The markers of CSCs in meningiomas include CD133, Sox2, nestin, and Frizzled-9 85-88. Baeesa SS *et al.* 84 reported a case of a Grade 1 meningioma that underwent MT into a Grade 3 meningioma, accompanied by extracranial metastasis. The authors observed that prior to the onset of metastasis, the tumor displayed CSC markers, the expression of which increased in the metastatic tissue. Additionally, primary cell lines derived from the metastatic tissue exhibited greater drug resistance and reduced apoptotic capacity. Consequently, because meningiomas can undergo MT, it may be feasible to predict this occurrence through the detection of CSCs.

## Prognosis of malignant transformation

Recurrent meningiomas are biologically, clinically and pathologically more aggressive than primary meningiomas. Meningiomas with MT are usually recurrent tumors and undergo treatment failure, therefore, have similar characteristics as recurrent meningiomas. Previous studies had demonstrated with each successive tumor recurrence, the effectiveness of salvage treatment decreased 89, 90. In the study of Momin *et al.* the median PFS after salvage radiotherapy for the 1^st^ or 2^nd^ recurrent grade 2 meningiomas was 47 months compared to 16 months for the 3^rd^ or more recurrence (p=0.003) 90. Prior radiotherapy also predicted poor prognosis. In the radiotherapy-naïve group, the 1-, 3- and 5-year PFS after salvage radiotherapy for recurrent grade 2 meningiomas were 96.3%, 65.8% and 44.2%, compared to 67.5%, 45.4% and 23.3% in the re-radiotherapy group (p=0.0084) 90. Therefore, many studies had demonstrated secondary meningiomas had poor tumor control rates 28, 91-95 and overall survival rates 26, 28, 49, 92, 94 than primary meningiomas. While some studies found secondary meningiomas were not significantly associated with poor tumor control rates 89, 96-98 and overall survival rates 48, 95-97. Table 1 summaries selected studies on prognosis of secondary meningiomas. In the secondary anaplastic meningiomas, Peyre *et al.* also found TERT mutations had a worse impact on the PFS 49. Due to the limited number and small proportion of secondary meningiomas reported in the retrospective studies, the prognosis of secondary meningiomas need to be further investigated through high quality clinical researches.

## The relationship between radiotherapy and malignant transformation

Radiotherapy has been established as an alternative therapy to surgery, adjuvant or salvage treatment after surgical resection for meningiomas. A major concern is that whether radiotherapy can induce MT of meningiomas. Erroneous repair of DNA damage after radiotherapy can result in gross genomic rearrangement, which can lead to genomic instability, resistance to therapy, and tumorigenesis 99. Meningiomas previously treated with adjuvant radiotherapy exhibit a significantly higher frequency of copy number alterations than radiation-naïve or radiation-induced meningiomas 100. The implanted intracranial tumors formed by irradiated meningioma cell were found to be metastatic with secondary centers along the spinal cord, indicating higher aggressiveness, while the tumors formed by untreated meningioma cells were localized to the brain and did not show any morphological features, indicating less aggressive behavior 101. These may be the mechanisms of MT induced by radiotherapy. In grade II IDH-mutant gliomas, chemotherapy and radiotherapy significantly increased malignant transformation rate per cell by 1.8 to 2.8 times compared with before treatment 102. So far, more than one million patients have undergone stereotactic radiosurgery 103, rare individual case reports were associating stereotactic radiosurgery with MT into higher-grade meningiomas 104, 105 and other tumors 106-108. It had been reported that radiotherapy can also induce the development of meningiomas. Meningiomas are the most common radiation-induced tumors after cranio-spinal radiotherapy, 109 with a 1/8 risk of developing radiation-induced meningiomas by the age of 40 110-112. Radiation-induced meningiomas were clinically aggressive, probably to be grade 2 meningiomas at first surgical resection (43.6%) and to progress after surgical resection (41%) 113. Furthermore, second malignancies can be induced by previous radiotherapy. It was reported the 5- and 15-year probability to develop second tumors based on histopathology in or near the first radiotherapy area, after intermediate or high radiation doses, was 0.5% and 2.2%, respectively 114. The overall median latency of second tumors was 7.4 years (1-42 years) 114. These above studies support the view that radiotherapy can induce MT of meningiomas. However, even without radiotherapy, meningioma can develop MT similar to gliomas, when occur recurrence after surgical resection. A systematic review and meta-analysis 22 did not found evidence that radiosurgery increased the risk of MT of grade 1 meningiomas. These might be due to the grade 1 meningiomas treated with radiosurgery were more frequently located in the skull base, and less frequently treated with salvage surgery, leading to an unknown WHO classification at progression. It was difficult to compare the incidence rate of MT between surgery and radiotherapy group. Therefore, further study is needed to confirm the impact of radiotherapy on the incidence rate of malignant transformation.

Another major concern is that whether radiotherapy can prolong time to MT through long term control of meningiomas. Radiosurgery had demonstrated efficacy and safety for treating benign meningiomas even in the medium to long term 115. It was reported WHO grade 1 meningiomas treated with radiosurgery had a long-term PFS ranging from 85% to 100% (median, 89%), and from 53% to 100% (median 85%) at 5 and 10 years respectively 116. For postsurgical residual and recurrent WHO grade 2 meningiomas, radiotherapy is recommended 3. Radiotherapy is a safe and effective treatment method for residual or recurrent grade 2 meningiomas. 117, 118 Sun *et al.* 117 reported SRS/EBRT had 2- and 5-year actuarial locoregional control rate of 91%/88% and 71%/69%, respectively. Aboukais *et al.* 118 also demonstrated the 1-, 2-, and 3-year actuarial local control rates and regional control rates for delayed progression after resection for grade 2 meningiomas were 75%, 52%, 40%, and 75%, 48%, 33%, respectively. Based on the efficacy of radiotherapy for meningiomas, it seems that time to MT could be prolonged. In IDH-mutant lower-grade gliomas (grade 2 or 3), radiotherapy is associated with delayed MT, the time to MT was 58.4 months in patients treated with radiotherapy compared with 32.6 months in patients without radiotherapy 119. In the study of Nakasu *et al.*, 22 time to MT was significantly longer in grade 1 meningiomas treated with radiotherapy before MT than in those who did not receive radiotherapy in univariate analyses, however, radiotherapy was not significantly associated with time to MT in multivariate analyses. These may be ascribed to high proportion of skull base meningiomas and low proportion of salvage surgery after radiotherapy in the patients who receive radiotherapy.

## Conclusions

Meningioma is the most common tumor in CNS tumors. WHO classification has been widely used for predicting tumor recurrence. With the continuous deepening of research on meningioma and the revision of WHO classification every time, the proportion of WHO grade 2 and grade 3 changes dynamically. MT is one of the main reasons leading to treatment failure of meningiomas. In this review, we review the incidence of MT of meningiomas. Due to the higher recurrent rate and more aggressive behavior, the incidence of MT of grade 2 meningiomas is likely to be higher than benign meningiomas. Time to MT of grade 2 meningiomas seemed to be shorter than MT of grade 1 meningiomas. Several risk factors may be associated with MT, including non-skull base location, high mitotic Index, a larger primary tumor size, shorter recurrence time interval and male. Potential molecular mechanisms of MT include chromosomal abnormalities, genomic alterations. Secondary meningiomas are usually recurrent tumors and undergo treatment failure. Therefore, they may have poor tumor control rates and overall survival rates than primary meningiomas. Besides, the role of radiotherapy in MT of meningiomas is unclear. Major concerns are whether radiotherapy can induce MT of meningiomas, and whether radiotherapy can prolong time to MT through long term control of meningiomas. This review summarizes the MT of meningiomas, and may provide the direction for further study of meningiomas.

## Figures and Tables

**Table 1:** Selected studies on prognosis of secondary meningiomas

Study	Patients, n	II^ary^ tumor, n(%)	WHO grade	Time to MT	Tumor control and risk factors		OS and risk factors	
Wang *et al.* 2019 91	263	31 (11.8)	2	NA	Median PFS: 2.3y	Tumor size≥41.5mm Extent of resection MIB-1>10 II^ary^ meningiomas	NA	NA
Li *et al.* 2019 92	302	52 (17.2)	2	Median: 3.2y	5-y RFS: 47.7%	KPS≥80 Invasiveness II^ary^ meningiomas	5-y OS:78.8%	KPS≥80 PTE Supratentorial II^ary^ meningiomas
Chen *et al.* 2018 89	65	10 (15.4)	2	NA	3-y LFFR:42%	Multifocal local recurrence	Median OS: 4.3y	NA
Champeaux *et al.* 2017 93	215	18 (8.4)	2	Median: 5.7y	5-y RFS: 82%	Simpson resection I/II Ki-67 II^ary^ meningiomas	Median OS: 11.5y	NA
Champeaux *et al.* 2016 94	194	31 (16.0)	2	Median: 5.7y	5y-RFS: 71.6%	Simpson grade II^ary^ meningiomas	5y-OS: 83.2%	Age II^ary^ meningiomas
Zhao *et al.* 2015 95	89	11 (12.4)	2	NA	5y-PFS: 67.5%	Symptom II^ary^ meningiomas	5y-OS: 89.1%	KPS score
Maier *et al.* 2022 98	51	24 (47.1)	3	Median: 5.5 y	NA	NA	Median OS: 4.2 y	NA
Champeaux *et al.* 2019 26	178	76 (42.7)	3	Medan: 7.5 y	NA	NA	5-y OS: 40%	Age at MM surgery Completeness of resection Adjuvant RT II^ary^ meningiomas
Peyre *et al.* 2018 49	57	29 (50.8)	3	Median: 4.6 y	NA	NA	5-y OS: 10%	Mitotic index II^ary^ meningiomas
Zhao *et al.* 2015 95	37	23 (62.2)	3	NA	5-y PFS: 12.1%	II^ary^ meningiomas	5-y OS: 19.9%	Multi-occupation
Moliterno *et al.* 2015 48	37	14 (37.8)	3	NA	NA	NA	5-y OS: 27.9%	GTR at 1^st^ surgery Convexity or parasagittal location
Yang *et al.* 2008 28	74	20 (27.0)	2/3	Median: 5.8y (I-II); Median: 7.4y (I-III) Median: 3.3y (II-III)	10-y DFS:87.1% (II); 5-y RFS:29% (III)	Brain invasion Adjuvant RT Extent of resection P53 expression II^ary^ meningiomas	10-y OS: 89.6% (II); 5-y OS:35% (III)	Brain invasion Adjuvant RT Extent of resection P53 expression II^ary^ meningiomas
Pasquier *et al.* 2008 96	119	16 (13.4)	2/3	Median 2.8±5y (I-II/III)	5-y DFS: 58%; 10-y DFS:48%	High mitotic rate	5-y OS: 65%; 10-y OS: 51%	Age > 60y High mitotic rate
Ferraro *et al.* 2014 97	35	3 (8.6)	2/3	NA	3-y PFS: 65%	Grade III tumors	3-y OS: 78%	Grade III tumors

Abbreviations: NA, not available; OS, overall survival, IQR, interquartile range; PFS, progression-free survival; RFS, recurrence-free survival; LFFR, local freedom from recurrence; DFS, disease free survival; PTE, peritumoral edema; MT, malignant transformation; II^ary^ meningiomas, secondary meningiom
